# Interaction between DNA and Trimethyl-Ammonium Bromides with Different Alkyl Chain Lengths

**DOI:** 10.1155/2014/863049

**Published:** 2014-01-16

**Authors:** Chao Cheng, Shi-Yong Ran

**Affiliations:** Department of Physics, Wenzhou University, Wenzhou 325035, China

## Abstract

The interaction between **λ**—DNA and cationic surfactants with varying alkyl chain lengths was investigated. By dynamic light scattering method, the trimethyl-ammonium bromides-DNA complex formation was shown to be dependent on the length of the surfactant's alkyl chain. For surfactants with sufficient long alkyl chain (CTAB, TTAB, DTAB), the compacted particles exist with a size of ~60–110 nm at low surfactant concentrations. In contrast, high concentration of surfactants leads to aggregates with increased sizes. Atomic force microscope scanning also supports the above observation. Zeta potential measurements show that the potential of the particles decreases with the increase of surfactant concentration (CTAB, TTAB, DTAB), which contributes much to the coagulation of the particles. For OTAB, the surfactant with the shortest chain in this study, it cannot fully neutralize the charges of DNA molecules; consequently, the complex is looser than other surfactant-DNA structures.

## 1. Introduction

Cationic surfactants, a kind of agent to induce DNA condensation [1], have been used in DNA extraction [2, 3], purification [4], enhancing the fluorescence intensity [5], and have potential uses in gene delivery [6, 7]. Because of its technological and biomedical importance, extensive studies have been carried out to elucidate the mechanism of DNA-surfactant interaction [8, 9]. It is commonly believed that hydrophobic force contributes much to the binding of surfactant molecules to the DNA molecules. The induced DNA compaction can occur well below the surfactant's critical micelle concentration because the DNA chain can act as a backbone for surfactants to aggregate. The process was displayed to be a discrete all-or-none type transition at the single DNA molecule level and to be a continuous transition at the DNA ensemble level [10]. Many factors, including surfactant concentration, incubation time, salt, temperature, DNA conformation, surfactant's chain length, and surfactant headgroup, affect the interaction between surfactant and DNA [11–16].

With the advantage of imaging DNA molecules directly, atomic force microscopy (AFM) [13, 17], fluorescence microscopy (FM) [10, 18–21], and electronic microscopy (EM) [22, 23] have been used to study the conformation of DNA-surfactant complex. Single molecule methods, which can monitor the process of DNA-surfactant interaction, are also useful tools to reveal the mechanism and the complex's structure [13, 24].

Dynamic light scattering (DLS) method [25], another useful technique, can detect the changes of size distribution of DNA-surfactant complexes by measuring their Brownian motions. In this way, the coil-globule transition of DNA molecules can be monitored in bulk directly. Several studies have used DLS to study the interaction between DNA and cationic surfactants [26–30] or other agents [31–34]. For example, Dias et al. studied the compaction and aggregation of DNA induced by CTAB using DLS [27]. Using this method, the compacted DNA molecules could be monitored by the appearance of a band with low hydrodynamic radius and by the decrease in the intensity of the peak corresponding to extended DNA molecules. They also found that at intermediate surfactant concentrations, compacted conformation coexists with extended one, which agrees well with the fluorescence microscopy observation. Marchetti et al. found that the complexation process reaches a maximum when the number of surfactant (CTAB or DDAO) and DNA phosphate groups is equal. The compacted DNA molecules were also found to have a higher thermal stability than the elongated ones [28].

Despite the extensive literature to investigate the DNA-surfactant interaction, this issue still needs more investigation. For example, though previous studies have concluded that high concentrations of cationic surfactants lead to aggregation, less experimental research elucidated why the complexes tend to aggregate and finally have larger sizes. Moreover, how the length of the surfactant affects the DNA conformation needs to be clarified further.

**Figure 1:**
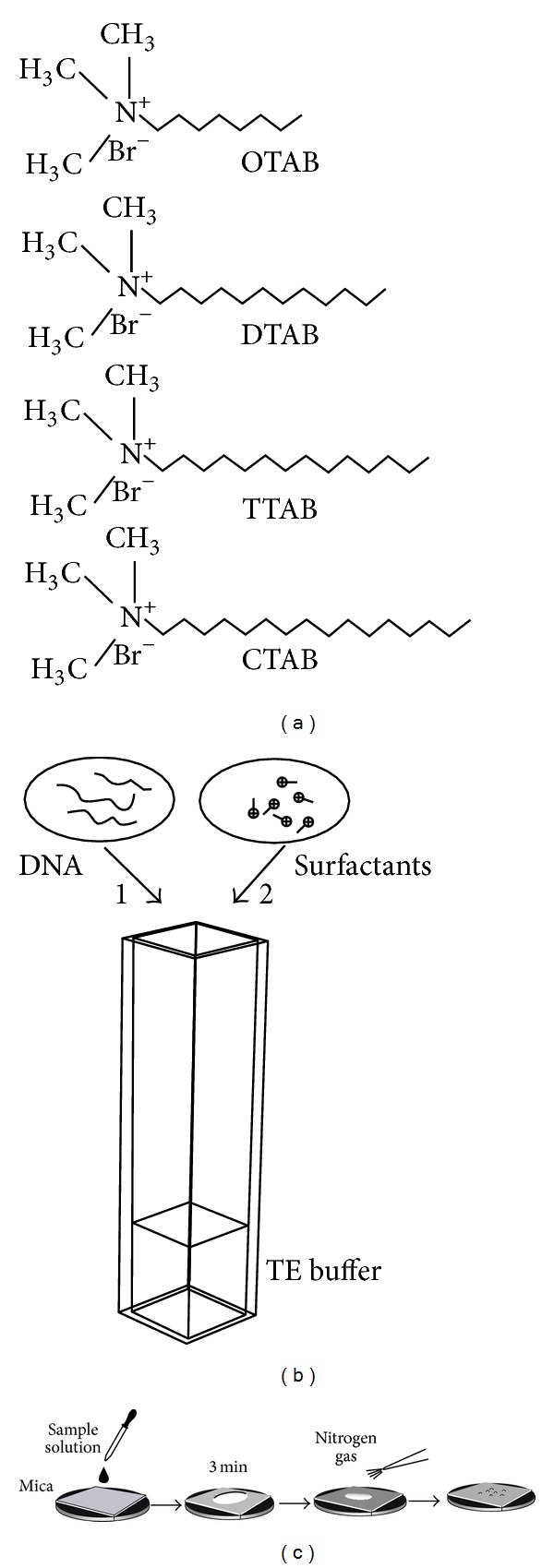
(a) Chemical structures of the surfactants used in the present research. The cationic trimethyl-ammonium surfactant has *n* = 8, 12, 14, and 16 carbon atoms in the hydrophobic tail, respectively; (b) schematic of the DNA-surfactant solution preparation. Firstly, 1.6 *μ*L DNA (stock concentration 0.5 *μ*g/*μ*L) was loaded into a capillary cell, then the surfactant solution was added; (c) the procedure of depositing sample on mica for AFM imaging.

In the present study, we aim to provide more clues on the previous problems by studying the interaction between a long-chain DNA (*λ*-DNA) and cationic surfactants with varying lengths [octyl-trimethyl-ammonium bromide (OTAB), dodecyl-trimethyl-ammonium bromide (DTAB), tetradecyl-trimethyl-ammonium bromide (TTAB), and cetyl-trimethyl-ammonium bromide (CTAB), Figure 1(a)]. DLS method was used to measure the size distribution. The zeta potentials of the condensed DNA particles were characterized under varying surfactant concentrations and chain lengths. As a complementary method, AFM was used to obtain the morphologies of the corresponding particles.

## 2. Material and Methods

### 2.1. Materials

OTAB, DTAB, TTAB, and CTAB (Figure 1(a)) were purchased from sigma and used without further purification. Bacterial *λ*-DNA (0.5 *μ*g/*μ*L, 48 500 bp) was purchased from New England Biolabs. 1x TE buffer (10 mM Tris-Cl + 1 mM EDTA, PH 8.0) was used to dissolve the surfactants. Distilled water obtained from a Milli-Q system was used in all sample preparations.

### 2.2. DLS and Zeta Potential Measurements

A Malvern Zetasizer Nano-ZS90 instrument was used for particle size and zeta potential measurements. The light source is a He-Ne laser with a wavelength of 633 nm. The laser power is automatically attenuated in order to make the count rate from the sample is within acceptable limits. Clear disposable capillary cells were used. Firstly, the cell was loaded with 0.8 mL diluted DNA solution (1 ng/*μ*L in 1x TE buffer). The concentrated surfactant solutions (10 mM for CTAB, 50 mM for TTAB, and 100 mM for DTAB and OTAB) were injected to the above DNA solutions to reach the final surfactant concentrations for study (Figure 1(b)). Every sample was incubated overnight to make sure the system reached a thermodynamically stable state. The sample was placed in a thermostated bath maintained at 25°C. The scattered light was detected at an angle of 90°.

### 2.3. AFM Imaging

A SPM-9600 system (Shimadzu, Kyoto, Japan) was used for scanning. A probe with a spring constant of 42 N/m and a resonance frequency of 320 kHz was used. All samples were scanned in the tapping mode at an ambient condition. All presented images were flattened to improve the contrast grade. The imaging was performed at a scan size of 5 × 5 *μ*m. All displayed images are height ones. For sample preparation, 10 *μ*L incubated solution was dropped onto a freshly cleaved mica surface and incubated for 3 min at room temperature. The surface was then dried with a gentle nitrogen gas flow. After drying, the samples were kept in a desiccator ready for scan (Figure 1(c)).

## 3. Results

### 3.1. Size Distribution and Particle Size Measurements

**Figure 2:**
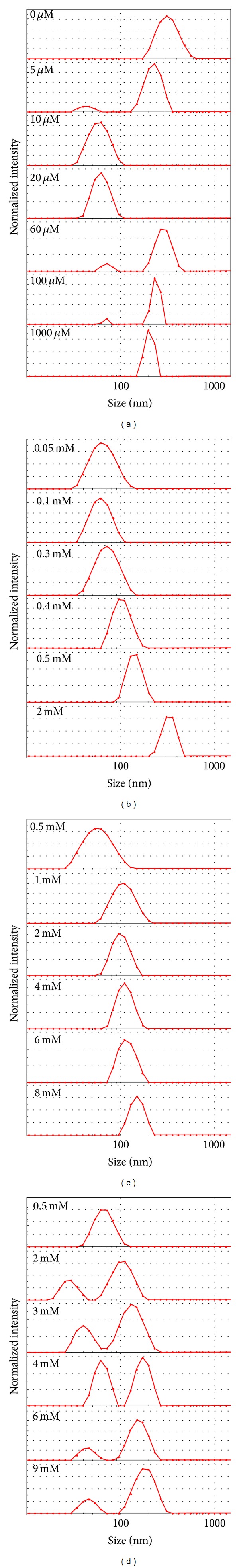
Typical intensity weighted distribution curves in the presence of different concentrations of surfactants; (a) CTAB, from top to bottom the concentrations are 0, 5, 10, 20, 60, 100, and 1000 *μ*M; (b) TTAB, from top to bottom the concentrations are 0.05, 0.1, 0.3, 0.4, 0.5, and 2 mM; (c) DTAB, from top to bottom the concentrations are 0.5, 1, 2, 4, 6, and 8 mM; (d) OTAB, from top to bottom the concentrations are 0.5, 2, 3, 4, 6, and 9 mM.

Firstly, we performed DLS measurements of the DNA solution. The concentration of the DNA solution was 1 ng/*μ*L. A representative intensity weighted size distribution of the DNA solution is shown in the top curve of Figure 2(a) with a peak showing a typical hydrodynamics radius of around 300 nm. This peak reflects the translational diffusion of DNA. However, it should be noted that the DLS measurement of the DNA solution in our study was not repeatable enough. Sometimes it exhibited more peaks with smaller radius, which may correspond to the internal dynamics of the DNA molecules. This should be attributed to the DNA molecule exhibiting a random coil conformation instead of a compacted spherical particle conformation.

The DNA samples with varying concentrations of surfactants added and incubated were then measured by DLS to obtain their size distribution curves. Typical intensity weighted distribution curves in the presence of different concentrations of surfactants are shown in Figure 2. For a CTAB concentration of 5 *μ*M, two peaks with a hydrodynamics radius of 42.6 nm and the other 223.4 nm appeared; this observation suggests the compacted DNA molecules coexist with DNA coils, as revealed by previous studies. Increasing the CTAB concentration leads to the disappearance of the larger-radius peak. However, as the concentration of CTAB increased to 60 *μ*M, a peak with a hydrodynamic radius of 384.1 mm is observed. Meanwhile, the peak with smaller radius is still detectable. With further increasing of the CTAB concentration, the magnitude of the peak with larger radius increases, while the intensity of the smaller-radius peak decreases. At 1 mM CTAB, only one peak with a radius of 203.2 nm is presented (Figure 2(a)).

The appearance of larger particles at CTAB concentrations above 60 *μ*M indicates the aggregation of CTAB-DNA particles. The coexistence of small compacted particles and larger aggregates, similar to the concomitance of coil chains and compacted particles elucidated by FM study, has not revealed before as far as we know. Then, whether decreasing the length of the surfactant chain leads to similar results is of concern.

As shorter-chain surfactants, TTAB and DTAB, can also cause DNA compaction and larger particle aggregates. It can be seen that increasing concentration also leads to peaks with increased sizes. In our study, the surfactant concentrations at which a considerable increase of the particle size occurred are 1 mM for TTAB and 8 mM for DTAB. However, no curves reflect the coexistence of two peaks indicating the smaller and larger particles (Figures 2(b) and 2(c)).

Interestingly, the bimodal size distributions appear again for the surfactant with the shortest chain, OTAB. One peak has a smaller radius, while the other shows a larger size (Figure 2(d)). Nevertheless, both cannot be attributed to the presence of compacted particles similar to that observed in the CTAB-DNA system, which can be verified by the following AFM characterization.

**Figure 3:**
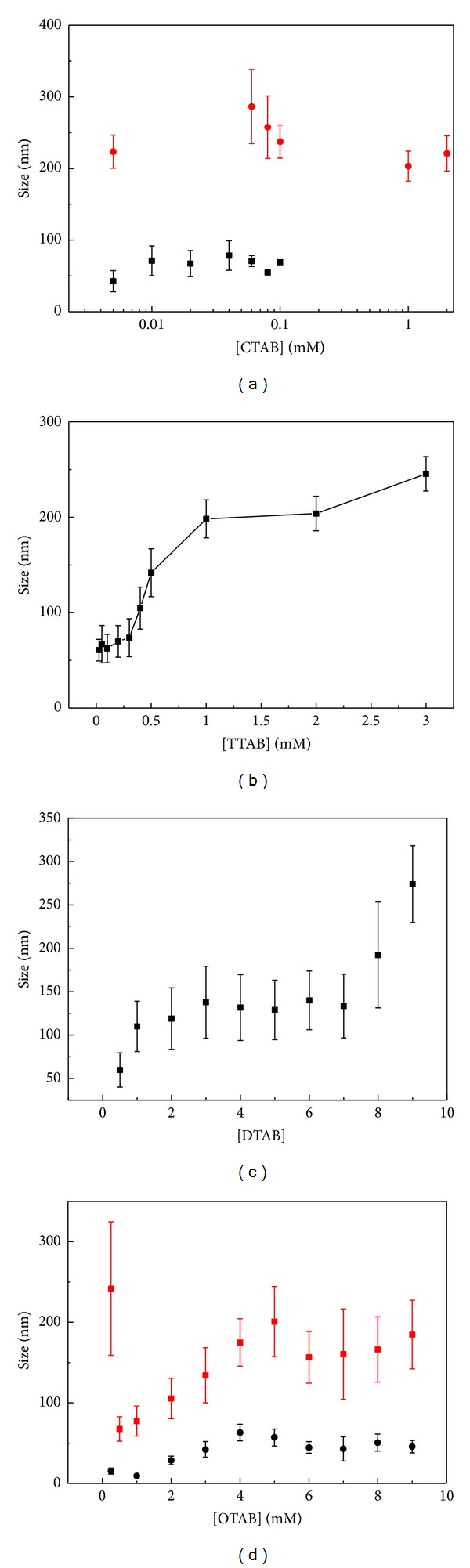
The hydrodynamic radius of the DNA molecules obtained from the size distribution curves as a function of surfactant concentration. Note that the error bars were deduce from the full width at half maximum in the DLS curves. (a) CTAB; (b) TTAB; (c) DTAB; (d) OTAB.

Figure 3 shows the hydrodynamic radius of the particles obtained from the size distribution curves as a function of surfactant concentration. It can be concluded that the longer the surfactant's chain, the less surfactant molecules are needed to induce DNA condensation, so does the critical concentration at which a considerable increase of the particle size occurs.

### 3.2. Zeta Potential Measurements

**Figure 4:**
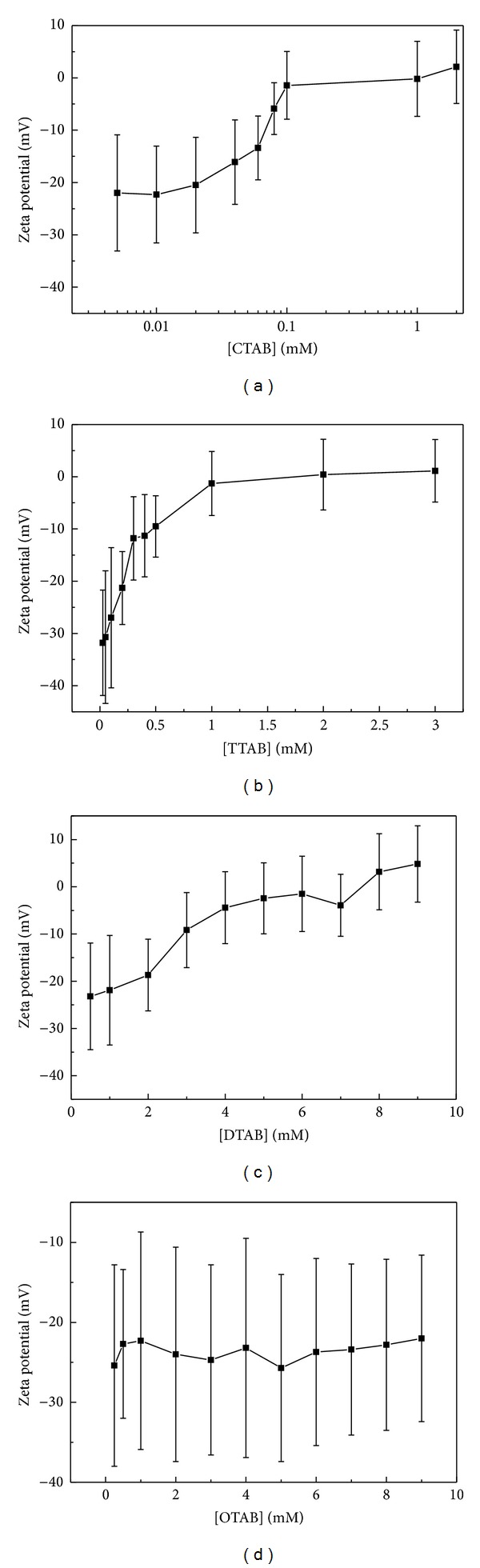
The zeta potential measurements as a function of surfactant concentration. The error bars refer to the full widths at half maximum in the measured curves. (a) CTAB; (b) TTAB; (c) DTAB; (d) OTAB.

Figure 4 shows the zeta potential as a function of surfactant concentration. For the CTAB-DNA solution, the zeta potential increases sharply from the original −22 mV to −1.45 mV with the increasing CTAB concentration from 5 *μ*M to 100 *μ*M. Above 100 *μ*M, it remains to be around 0 mV with no significant changes. For the TTAB-DNA solution, the considerable change of the zeta potential occurs in the region between 50 *μ*M and 1 mM, with an increase from −30.7 mV to −1.3 mV. For the DTAB-DNA solution, the zeta potential increases smoothly from −23.2 mV to 4.84 mV as the concentration increases from 0.5 mM to 9 mM. Surprisingly, the OTAB-DNA solution shows no significant change of the zeta potential. Although the concentration increased up to 9 mM, the zeta potential remains to be around −23 mV, which is far below 0 mV.

### 3.3. AFM Observation

The DLS results indicate that the surfactant-DNA particles have smaller sizes under low surfactant concentrations, while have larger ones under high surfactant concentrations. To give visual verification, AFM scanning was performed.

**Figure 5:**
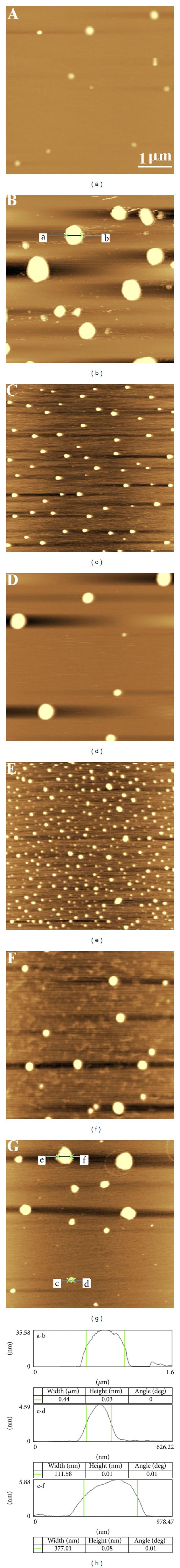
Typical scanning images of DNA-surfactant complexes with the same scale shown in Figure 5(a); (a)-(b) the CTAB-DNA complexes with [CTAB] = 10 and 100 *μ*M, respectively; (c)-(d) the TTAB-DNA complexes with [TTAB] = 0.3 and 1 mM, respectively; (e)-(f) the DTAB-DNA complexes with [DTAB] = 2 and 9 mM, respectively; (g) the OTAB-DNA complexes at [OTAB] = 4 mM; (h) the morphology analysis of the particles presented in Figures 5(b) and 5(g). The analyzed particles in Figure 5(g) present a typical height of ~5 nm, which is far less than the height of the particle (35.58 nm) in Figure 5(b).

The representative results are shown in Figure 5. The deduced particles with smaller and larger sizes in DLS study can be found in the corresponding representative AFM characterizations. Generally, all the condensed particles except OTAB-DNA complexes exhibit typical globular conformations. The OTAB-DNA complexes have lower heights (<6 nm) compared with the conformations of other surfactant-DNA particles, as shown by the morphology analysis in Figure 5(h). This indicates that the OTAB-DNA structure is loose and OTAB cannot compact DNA with high efficiency. Moreover, the AFM scanning of the OTAB-DNA complexes suggests the coexistence of particles with large sizes and little ones, which is consistent with the bimodal size distribution in DLS measurements.

## 4. Discussion

### 4.1. The Condensing Ability of the Surfactants

The DLS results and zeta potential measurements both suggest that the compaction efficiency of surfactant increases with the surfactant chain length. To be more specific, achieving a zeta potential of ~10 mV needs the addition of ~60 *μ*M CTAB, ~0.5 mM TTAB and ~3 mM DTAB, respectively. While for OTAB, increasing the concentration does not alter the potential significantly. AFM scanning also concludes that OTAB cannot cause a compact complex structure like other surfactants.

Though TTAB and DTAB can cause DNA compaction like CTAB, they cannot produce particles with bimodal distributed sizes. Such phenomenon may result from the high compacting efficiency of CTAB. The increase of particle size occurs at a relatively low CTAB concentration, which indicates that less surfactant molecules contribute to aggregation compared with the cases of TTAB and DTAB. Due to the lack of sufficient CTAB molecules associating with smaller-size particles, the aggregation process may stop with a considerable amount of particles do not change in their sizes. Consequently, the size distribution curves were shown to have double peaks. And in the presence of enough CTAB molecules, the bimodal distribution would disappear, as shown in the previous section.

Why OTAB cannot compact DNA into a tightly condensed structure need to be elucidated further. It is known that elastic, hydrophobic, and electrostatic factors contribute to DNA packaging. As to cationic surfactants, its hydrophobic forces and screening effects are the main terms to drive DNA to condense, while the elastic rigidity and the electrostatic repulsion between the DNA chain resist DNA compaction. The competition between the driving terms and the resisting ones determines the final conformation of DNA-surfactant complexes. If hydrophobic force overcomes the resisting terms, spherical compacted particles are the favorable conformations, which are the cases for the surfactants with sufficient long chain (CTAB, TTAB, and DTAB). For short-chain surfactants such as OTAB, the hydrophobic force is not enough to surmount the resisting ones and consequently leads to less compact structures.

The results of the present work are consistent with the observation by Husale et al. [24]. They suggest different binding modes depending on the length of the surfactant chain based on the mechanical behaviors of the DNA-surfactant complexes. Short-chain surfactants (OTAB) could lie down on the DNA surface and do not cause DNA condensation. In contrast, long-chain surfactants could have their aliphatic tails pointing away from the DNA surface, which facilitate intermolecular interactions between surfactant molecules and then induce DNA condensation. Based on the present work, it can be concluded that the association between DNA and OTAB molecules is loose and cannot compact DNA efficiently. Despite that, OTAB do change the DNA conformation according to the AFM results. With the addition of OTAB molecules, DNA molecules adopt semi-compact structures (but not densely compact structures) instead of coil conformations.

### 4.2. Assessment of the Aggregation at High Surfactant Concentrations and the Zeta Potential Measurements

The aggregation between the particles, both revealed by the particle size measurements and AFM characterization, can be discussed in the field of colloid science. The magnitude of the zeta potential indicates the stability of the colloidal system. In the absence of surfactants, the DNA solution used in this study has a negative zeta potential of about −35 mV. With the addition of surfactants, the charges of the DNA are neutralized step by step, along with the decrease of the absolute value of the zeta potential. When DNA molecules are compacted into particles with small sizes under low surfactant concentrations, there exist considerable negative charges on them, which can prevent other charged particles from associating. In contrast, increasing surfactant concentration decreases the amount of the charges in the particles. Consequently, the electrostatic repelling forces between them are weakened and aggregation occurs.

The size distribution and zeta potential measurements suggest that the size of a particle depends on its charges. Next, we follow the procedures described in textbook to acquire the relation [35]. The net charge *q* of a particle with a radius of *R*_*s*_ is equal and opposite to the total charge in the double layer. If the particle is deemed as spherical, its charges can be evaluated by calculating the charges of the double layer:

(1) $$\begin{array}{c} q=-\int_{R_{s}}^{\infty }4\pi r^{2}\rho \cdot dr. \end{array}$$

Substituting Poission's Equation

(2) $$\begin{array}{c} \frac{1}{r^{2}}\frac{\partial }{\partial r}\left(r^{2}\frac{\partial \psi }{\partial \theta }\right)=-\frac{\rho }{\varepsilon }, \end{array}$$

into it gives

(3) q=4πε∫Rs∞ddr(r2dψdr)·dr=4πε(r2dψdr)Rs∞=−4πεRs2(dψdr)Rs,

where *ρ* is the charge density, *ψ* is the potential, *r* is the distance of any point in the double layer from the center of the particle, and *R*_*s*_ is the radius of the particle. The solution of the linearized Poission-Boltzmann Equation gives the relation between the potential *ψ* and the zeta potential *ζ*:

(4) $$\begin{array}{c} \psi =\frac{R_{s}\zeta }{r}\exp\left[-\frac{\left(r-R_{s}\right)}{\kappa^{-1}}\right]. \end{array}$$

**Figure 6:**
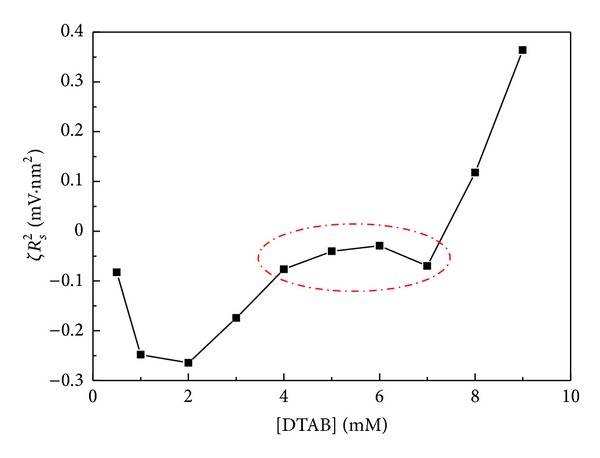
Plot of *ζR*_*s*_^2^ versus DTAB concentration combining the data of Figures 3(c) and 4(c).

Combining (3) and (4) leads to the result

(5) $$\begin{array}{c} q=4\pi \varepsilon \zeta R_{s}\left(1+\frac{R_{s}}{\kappa^{-1}}\right), \end{array}$$

where *κ* is the Debye-Hükel parameter and *κ* ^−1^ can be deemed as the “thickness” (imprecisely) of the double layer with a unit of length. In a 0.01 M solution of 1 : 1 electrolyte, *κ* ^−1^ has a typical value of 3.04 nm. The particle sizes in this study are generally larger than 50 nm; hence, *R*_*s*_/*κ* ^−1^ ≫ 1 and *q* = 4*πεζR*_*s*_(1 + *R*_*s*_/*κ* ^−1^) ≈ (4*πε*/*κ* ^−1^)*ζR*_*s*_^2^. If the dielectric constant of the solution and *κ* ^−1^ being approximately invariant in all DNA-surfactant systems, we can plot *ζR*_*s*_^2^ as a function of surfactant concentration to infer the variation tendency of the charges carried by the particles.  Figure 6 shows the trend of *ζR*_*s*_^2^ with the increase of DTAB concentration. The occurrence the circled plateau reflects the approximate invariance of the charges. Such a coarse analysis seems to indicate a delicate equilibrium between the zeta potential and the particle size. In other words, the particle tends to increase its size to counterbalance the increasing screening effects of the surfactant molecules.

On the other hand, the aggregation of the particles under high compacting agent concentration might be troublesome in gene delivery, because of the increased size leading to a high steric hindrance when passing through the cell membrane. In addition, it would be more difficult to release the DNA for therapy from such particles. Therefore, it is necessary to control the size of the particles if used in this field. In comparison, the aggregation will be helpful in DNA extraction or purification, because it facilitates the sedimentation process. Based on our investigation, it is shown that measuring the zeta potential of the particles is a convenient method to judge whether they will aggregate.

## 5. Conclusion

The surfactant-induced DNA condensation has been shown to be dependent on the length of the surfactant's alkyl chain. Surfactants with longer alkyl chain compact DNA molecules with fewer surfactant molecules. For surfactants with sufficient long alkyl chain (CTAB, TTAB, DTAB), the particles exist with a size of 60–110 nm at low surfactant concentrations. In contrast, high concentration of surfactants leads to larger aggregates. Zeta potential measurements show that the charges carried by the particles decreases with increasing surfactant concentration. It is the reduced electrostatic repulsion that caused the large aggregates. For OTAB, the surfactant with the shortest chain in this study, it was shown to be a low-efficiency compact agent, which cannot fully neutralize the charges of DNA molecules.
